# *In Vitro* Grown Sheep Preantral Follicles Yield Oocytes with Normal Nuclear-Epigenetic Maturation

**DOI:** 10.1371/journal.pone.0027550

**Published:** 2011-11-21

**Authors:** Barbara Barboni, Valentina Russo, Sandra Cecconi, Valentina Curini, Alessia Colosimo, Maria Luigia A. Garofalo, Giulia Capacchietti, Oriana Di Giacinto, Mauro Mattioli

**Affiliations:** 1 Department of Comparative Biomedical Science, University of Teramo, Teramo, Italy; 2 Department of Biomedical Sciences and Technologies, University of L'Aquila, L'Aquila, Italy; Brunel University, United Kingdom

## Abstract

**Background:**

Assisted reproductive technologies allow to utilize a limited number of fully grown oocytes despite the presence in the ovary of a large pool of meiotically incompetent gametes potentially able to produce live births. *In vitro* folliculogenesis could be useful to recruit these oocytes by promoting their growth and differentiation.

**Methodology/Principal Findings:**

*In vitro* folliculogenesis was performed starting from sheep preantral (PA) follicles to evaluate oocyte nuclear/epigenetic maturation. Chromatin configuration, quantification of global DNA methylation, and epigenetic remodelling enzymes were evaluated with immunocytochemistry, telomere elongation was assessed with the Q-FISH technique, while the DNA methylation status at the DMRs of maternally *IGF2R* and *BEGAIN*, and paternally *H19* methylated imprinted genes was determined by bisulfite sequencing and COBRA. Specifically, 70% of PA underwent early antrum (EA) differentiation and supported in culture oocyte global DNA methylation, telomere elongation, TERT and Dnmt3a redistribution thus mimicking the physiological events that involve the oocyte during the transition from secondary to tertiary follicle. Dnmt1 anticipated cytoplasmic translocation in *in vitro* grown oocytes did not impair global and single gene DNA methylation. Indeed, the *in vitro* grown oocytes acquired a methylation profile of *IGF2R* and *BEGAIN* compatible with the follicle/oocyte stage reached, and maintained an unmethylated status of *H19*. In addition, the percentage of oocytes displaying a condensed chromatin configuration resulted lower in *in vitro* grown oocytes, however, their ability to undergo meiosis and early embryo development after IVF and parthenogenetic activation was similar to that recorded in EA follicle *in vivo* grown oocytes.

**Conclusions/Significance:**

In conclusion, the *in vitro* folliculogenesis was able to support the intracellular/nuclear mechanisms leading the oocytes to acquire a meiotic and developmental competence. Thus, the *in vitro* culture may increase the availability of fertilizable oocytes in sheep, and become an *in vitro* translational model to investigate the mechanisms governing nuclear/epigenetic oocyte maturation.

## Introduction

The need to expand the availability of female gametes remains an important target of reproductive biotechnology. Using the current assisted reproductive technologies, it is possible to utilize a limited number of oocytes from antral follicles despite the presence of a large pool of gametes potentially able to produce live births. *In vitro* folliculogenesis could represent a useful technology to recruit, at least in part, these gametes by promoting their growth and differentiation. However, apart from the murine model, where *in vitro* production of fertilizable oocytes has reached high levels of efficiency [1], [2], in larger mammals this approach is still experimental. The difficulty of reproducing the process of folliculogenesis is related to the complexity of the molecular events controlling follicle/oocyte growth as well as to the long period of time required physiologically to produce a competent egg (from months to years, depending on the species) [3]. The oocyte, in fact, acquires its full meiotic and developmental competence at the end of complex processes affecting the ooplasm, where metabolic and regulative pathways controlling cell cycle are progressively established [4], and the nucleus, where profound structural and biochemical/epigenetic modifications occur [5]–[9]. In this context, the large-scale chromatin condensation described in several mammalian oocytes during the process of growth is correlated with the progressive repression of the oocyte-genome global transcription, which is required in order to reach a complete developmental potential [8]–[13]. At the same time, the occurrence of cytosine methylation produces in the oocyte a wide range of biological functions, including the reprogramming of the germ cell itself and the success of the early embryo-foetal development. However, even if the relevance of DNA methylation for embryo development and offspring health has been clearly demonstrated [14], [15], it is becoming evident that the maintenance of a correct epigenetic profile may be compromised by assisted reproductive technologies procedures [16]–[18].

Imprinting disorders correlated with abnormal establishment and/or maintenance of methylation patterns in oocyte/embryos can arise either after treatments of exogenous hormonal stimulations [18]–[21] or as a consequence of specific *in vitro* cultural conditions as medium composition and culture time [22], [23]. In particular, commercial available culture media display relevant differences in the levels of methyl donors (methionine, folates, choline, Vit B6 and 12 ecc.) thus providing a potential mechanism for inducing epigenetic changes during IVC techniques [24]–[28]. This last aspect may become particular relevant under *in vitro* folliculogenesis, when the oocyte defines its primary imprinting during a long cultural time [29]. In this contest, while Kerejean et al. [30] observed a dysregulation of the process with the loss and the gain of methylation at the *IGF2R* and *H19* locus, respectively, during early mice PA follicles culture, on the contrary, any influence on maternal primary imprinting was revealed by Anckaert E. group [26]–[28] even when the early PA follicles growth were reproduced under different *in vitro* cultural conditions. Actually, there are no evidences providing insight into the process of primary imprinting during sheep oocyte growth *in vitro*. However, epigenetic change in *IGF2R* has been definitively associated in sheep with the high incidence of fetal overgrowth syndrome that affect newborns obtained from IVC derived embryos [16].

Starting from these premises, the present research was designed to evaluate how the process of nuclear maturation occurs when the process of oocyte/follicle growth (from preantral, PA, to early antral, EA, follicle stage) is reproduced *in vitro* by applying a standard cultural condition adapted for the sheep animal model [31], [32]. In particular, the process of oocyte chromatin remodelling was analyzed on *in vitro* grown oocytes by studying nuclear events such as chromatin configuration, global DNA methylation, telomere elongation and the methylation status of an early and late imprinted gene previously identified in ovine oocytes (*IGF2R*, insulin-like growth factor 2 receptor; *BEGAIN*, brain-enriched guanylate kinase-associated protein 2B) [33]. In addition, the study of the methylation status of specific differentiated methylated regions (DMRs) have been extended to the *H19* [33]. These crucial nuclear events obtained *in vitro* were then compared with those displayed by oocytes that underwent growth under physiological conditions (EA and antral enclosed oocytes). To better understand the dynamism of these events leading to chromatin remodelling, in parallel, the ooplasm/nuclear distribution of the enzymes involved in the processes of telomere elongation (telomerase reverse transcriptase, TERT), and DNA methylation (DNA methyltransferase 1, Dnmt1, and DNA methyltransferase 3a, Dnmt3a) were documented on single cell. At the end of culture, the efficiency of the process of oocyte differentiation promoted under *in vitro* condition was tested by comparing oocyte meiotic and developmental competences after *in vitro* fertilization (IVF) and parthenogenetic activation. Therefore, this paper provides additional information on the potential/safety of follicle cultures and foster the development of a technique that could greatly increase oocytes availability for assisted reproduction. Beyond the practical relevance of growing oocyte *in vitro*, widening the interval of oogenesis that can reliably be reproduced *in vitro* offers great opportunities to investigate this crucial phase of gamete maturation that so heavily conditions the future of embryo/foetal development.

## Results

### FOLLICLE AND OOCYTE GROWTH IN CULTURE

**Figure 1:**
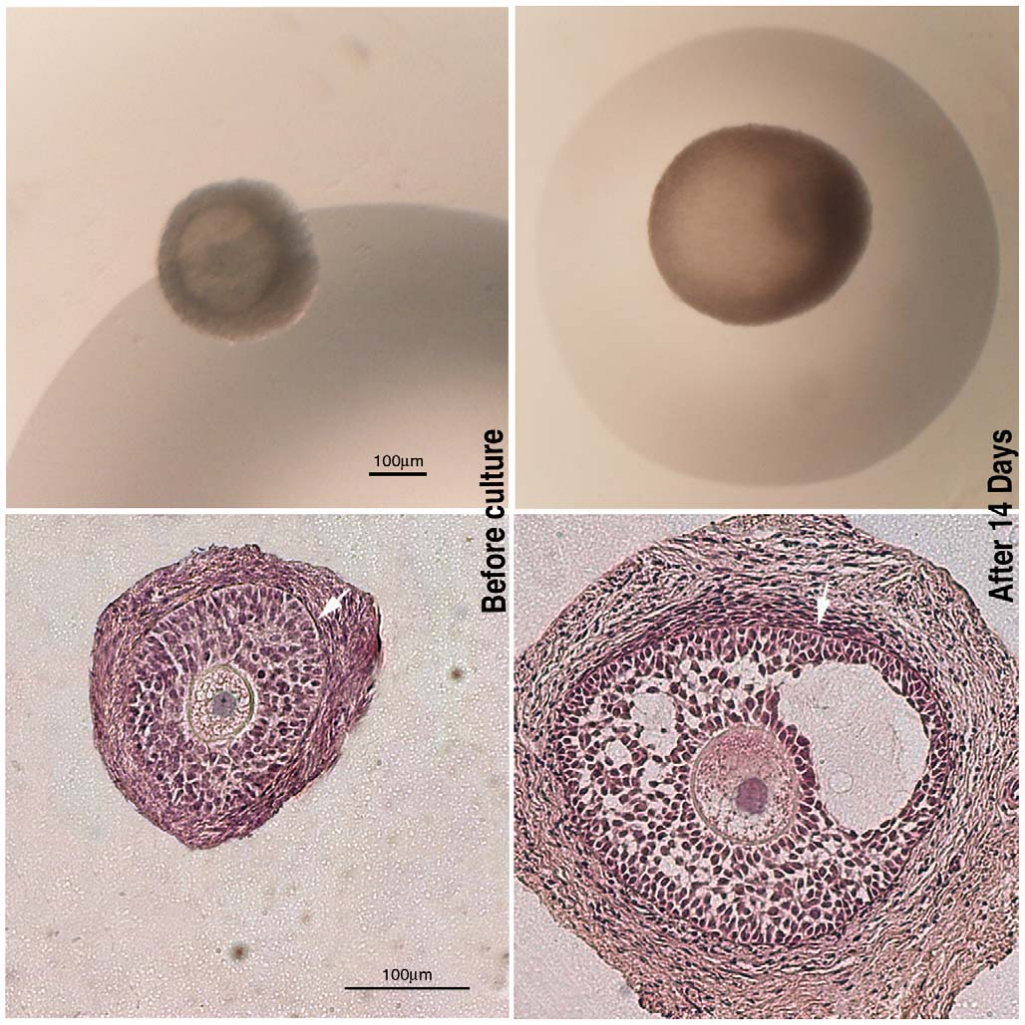
Representative images of the morphology of the follicles before and after the culture. On the left panels, examples of preantral (PA) follicles used for the culture and analyzed under a stereomicroscope (on the top) and after staining with haematoxylin/eosin (HE) (at the bottom). On the right panels, early antral (EA) follicles cultured for 14 days and analyzed under a stereomicroscope (on the top) and after staining with haematoxylin/eosin (at the bottom). Either in PA and EA follicles HE staining showed the presence of a compact layers of granulosa cells and a theca compartment with several fusiform cells (arrows).

PA follicles cultured *in vitro* for 14 days increased their diameter from 170±30 µm to 340±20 µm (*P*<0.01), and about 70% of them formed an early antral cavity becoming EA follicles (Fig. 1).

The germ cells isolated from these follicles significantly increased their diameter (from 75±10 to 110±5 µm; *P*<0.01), thus achieving a final size comparable to that of oocytes isolated from *in vivo* grown EA follicles (115±10 µm; *P*>0.05). The mean diameter of oocytes isolated from EA follicles resulted slightly lower than that recorded in fully grown oocytes isolated from antral or preovulatory follicles (120±5 µm; *P*>0.05).

### COMPARISON OF NUCLEAR REMODELLING BETWEEN *IN VITRO* AND *IN VIVO* GROWN OOCYTES

#### A. Chromatin configuration in germinal vesicle (gv) oocytes

**Figure 2:**
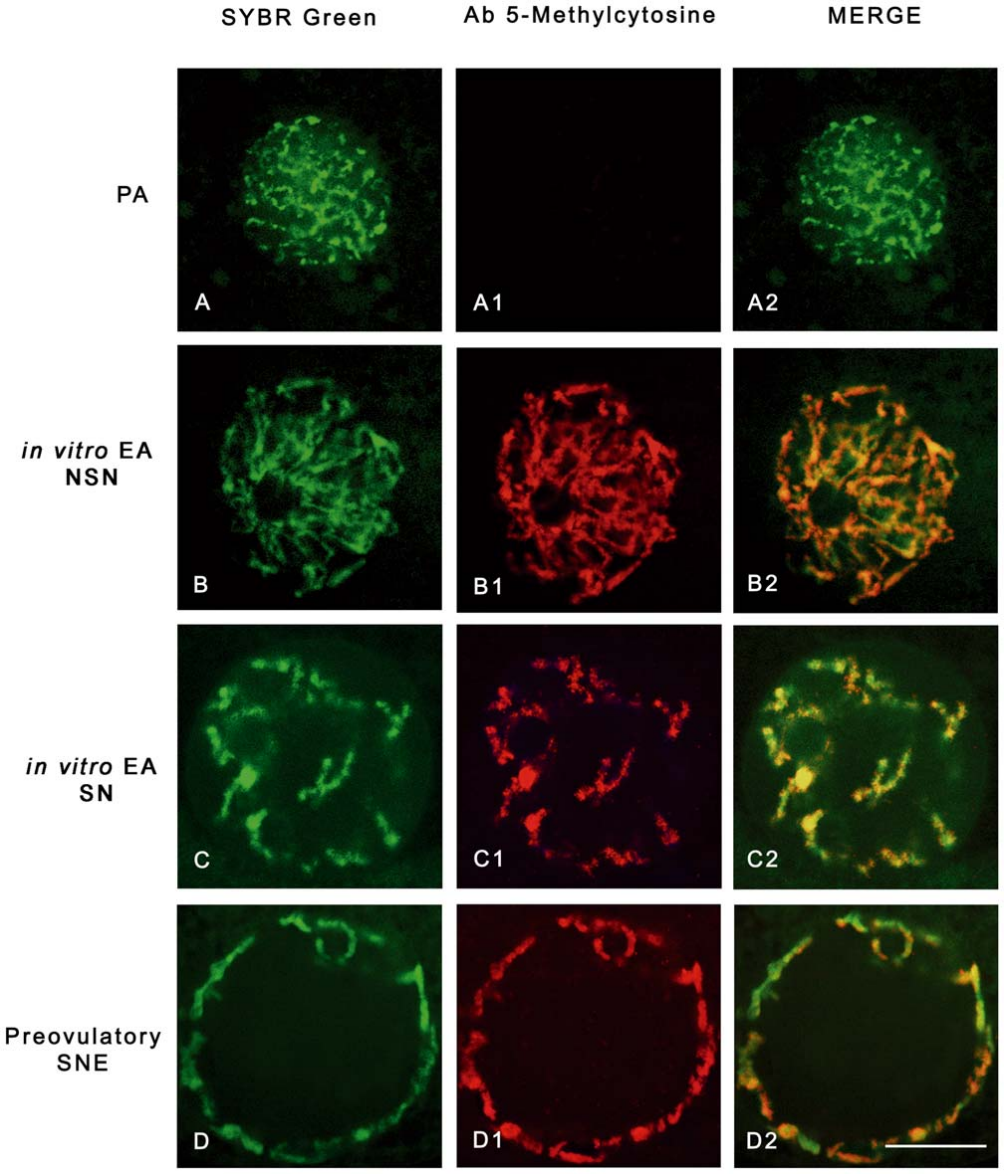
Chromatin remodelling and global DNA methylation in *in vitro* and *in vivo* grown oocytes. Digital images of sheep germinal vesicles (GVs) showing different chromatin configurations (left images), global DNA methylation patterns (middle images), and merged images (right images). Chromatin counterstaining was performed with SYBR Green 14/I (green), which detects double-stranded DNA, while global DNA methylation was analyzed with a 5-methylcytosine antibody (red). (**A**) A typical example of a GV germ cell obtained from a preantral (PA) follicle with a diffuse chromatin not surrounding the nucleolus (not surrounded nucleolus configuration: NSN) that did not display any immunostaining for 5-methylcytosine (A1,A2). (**B,C**) oocytes obtained from *in vitro* grown early antral (EA) follicles with either a NSN (B) or a surrounded nucleolus (SN) chromatin configuration (C), respectively. As observed in oocytes obtained from *in vivo* grown EA follicles, in both categories of *in vitro* grown oocytes the 5-methylcytosine immunopositivity indicates a comparable degree of global DNA methylation (NSN = B1,B2; SN = C1,C2). (**D**) GV oocyte isolated from a preovulatory follicle with a condensed chromatin localized surrounding the nucleolus and the nuclear envelope (SNE configuration) with a clear merged positivity with 5-methylcytosine antibody (D1,D2). Scale bar = 10 µm.

As summarized in Table 1, all the oocytes isolated from PA follicles displayed a highly dispersed chromatin within the nucleoplasm, defined as NSN (not surrounded nucleolus) configuration (Fig. 2). At the end of culture, 60% of oocytes retained this chromatin configuration, while the remaining developed a more condensed chromatin forming a typical ring around the nucleolus (surrounded nucleolus configuration: SN; Fig. 2). A significantly higher percentage of SN oocytes was recorded in *in vivo* grown EA follicles (65%; *P*<0.01). A more mature chromatin organization involved the oocytes isolated from preovulatory follicles where a highly condensed chromatin resulted distributed around the nucleolus and close to the nuclear envelope (SNE chromatin configuration; Fig. 2).

**Table 1:** Incidence of the different patterns of chromatin configuration in *in vitro* and *in vivo* grown oocytes.

Oocyte origin	NSN	SN	SNE
PA	100^a,A^	0^a,B^	0^a,B^
*in vivo* EA	35^b,A^	65^b,B^	0^a,C^
*in vitro* EA	60^c,A^	40^c,B^	0^a,C^
Preovulatory	0^d,A^	0^a,A^	100^b,B^

The percentages of chromatin configurations in oocytes isolated from PA, *in vivo* and *in vitro* grown EA and preovulatory follicles. Different superscripts denote values with highly significative (*P*<0.01) differences (χ2 test).

Data are expressed as percentage.

a,b,c,d = different superscripts denote differences among groups.

A,B,C = different superscripts denote differences within groups (NSN vs. SN vs. SNE).

#### B. Oocyte global dna methylation

**Figure 3:**
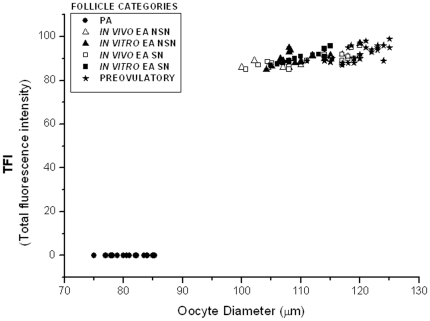
Global DNA methylation total fluorescence intensity vs. oocyte diameter. Comparative analysis of the global DNA methylation recorded as total fluorescence intensity (TFI) value in oocytes isolated from preantral (PA), *in vivo* and *in vitro* grown early antral (EA) and preovulatory follicles. The distribution of data revealed that global DNA methylation increased during the transition from PA to EA follicles, in parallel, with the increase of the oocyte diameter (r ranged between 0.694 and 0.933; *P*<0.05). The global DNA methylation reached a plateau in the oocytes isolated from tertiary follicles. The TFI emitted from each germinal vesicle was measured using the LaserPix software (Bio-Rad).

As shown in Figs. 2, 3, the levels of global DNA methylation significantly increased during follicle culture and with oocyte growth. Statistical analysis revealed that total fluorescence intensity (TFI) and oocyte diameter were strictly related to each others (r ranged between 0.694 and 0.933; *P*<0.05) without any significant difference between NSN and SN configuration (*P*>0.05). In fact, TFI values increased from no detectable levels of oocytes isolated from PA follicles to ∼90 in the germ cells collected from *in vitro* and *in vivo* grown EA follicles (91±6.7 and 88±7.4, respectively; *P*>0.05; Fig. 2). Moreover, TFI value did not further increase in fully grown oocytes isolated from preovulatory follicles (92±6.1; *P*>0.05).

#### C. Dna methyltranferase 1 and 3a intracellular localization

**Figure 4:**
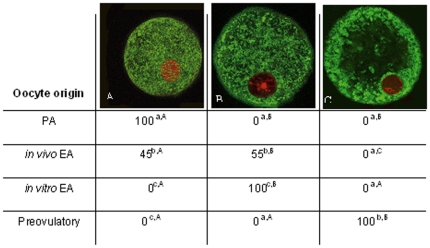
Dnmt1 oocyte distribution pattern during sheep *in vitro* and *in vivo* folliculogenesis. At the top of the figure are indicated the three major oocyte distribution patterns of the Dnmt1 during sheep *in vitro* and *in vivo* folliculogenesis, displaying the enzyme localized: A) both in the nucleus and the cytoplasm; B) widespread within the cytoplasm; C) in the subcortical and perinuclear region. Data are expressed as percentage. a,b,c = different superscripts denote differences among groups (*P*<0.01, χ2 test). A,B,C = different superscripts denote differences within groups (A vs. B vs. C) (*P*<0.01, χ2 test).

As summarized in Fig. 4, the oocytes isolated from PA follicles displayed the Dnmt1 protein distributed between nucleus and cytoplasm. The enzyme became cytoplasmatic in 100% of *in vitro* grown and 55% of *in vivo* grown oocytes isolated from EA follicles (*P*<0.01) independently of their chromatin configuration (data not shown). In fully grown oocytes isolated from preovulatory follicles the Dnmt1 appeared confined within the ooplasm distributed in the subcortical and perinuclear area.

**Figure 5:**
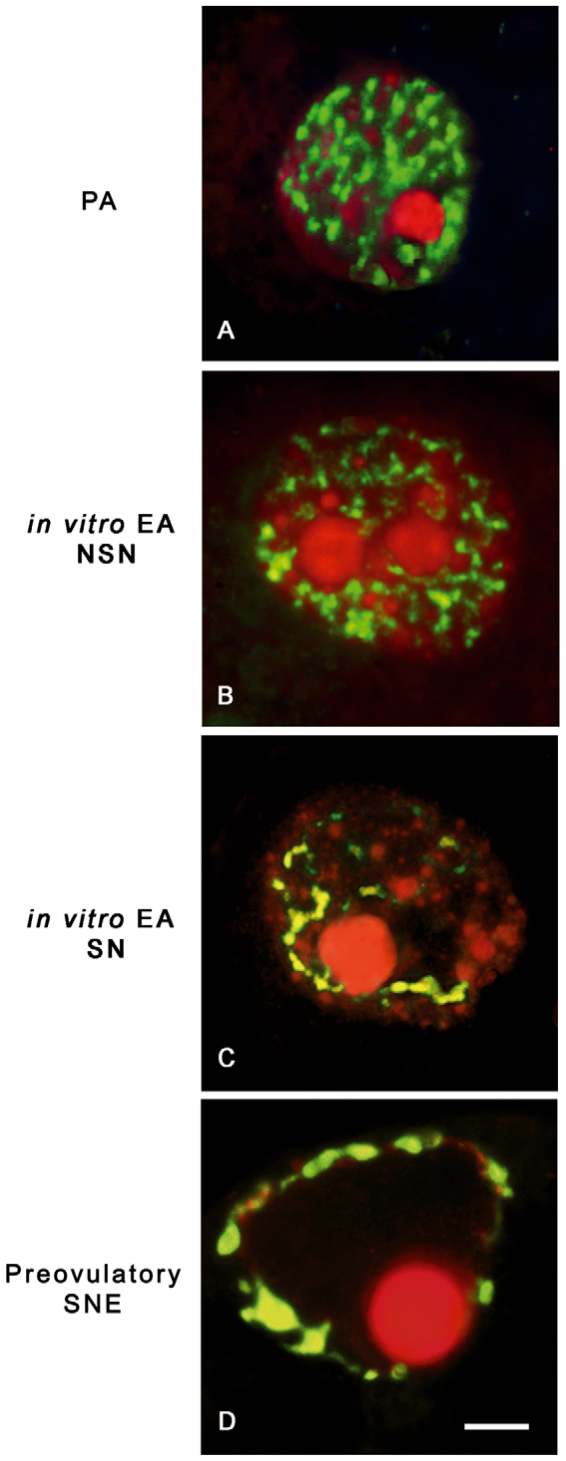
Dnmt3a nuclear distribution pattern during *in vitro* and *in vivo* oocyte growth. Examples of digital images showing Dnmt3a distribution pattern visualized with a Cy3 secondary antibody (red), while DNA was counterstained with SYBR Green 14/I (green). Dnmt3a maintained a nuclear localization in all *in vitro* and *in vivo* classes of germ cells analyzed. (**A,B**) Oocyte obtained from a preantral (PA) follicle (A) and from an *in vitro* grown oocyte isolated from an early antral (EA) follicle (B) with a not surrounded nucleolus (NSN) configuration: Dnmt3a is homogeneously distributed within the nucleus with a clustered arrangement in the vicinity of the DNA. (**C**) *In vitro* grown oocyte collected from an EA follicle which displays a surrounded nucleolus (SN) chromatin configuration: Dnmt3a is associated with DNA or gathered in clusters around it. (**D**) Oocyte isolated from a preovulatory follicle where Dnmt3a is strictly associated with the condensed chromatin. Scale bar = 5 µm.

By contrast, Dnmt3a maintained a nuclear localization in all classes of germ cells analyzed as showed in Fig. 5. The totality of NSN oocytes showed the Dnmt3a organized in clusters within the nucleus without any relation with the dispersed chromatin. Instead, Dnmt3a started to co-localize with the DNA of SN oocytes becoming strictly associated with the condensed chromatin in SNE oocytes isolated from preovulatory follicles.

#### D. Telomere sizing

**Figure 6:**
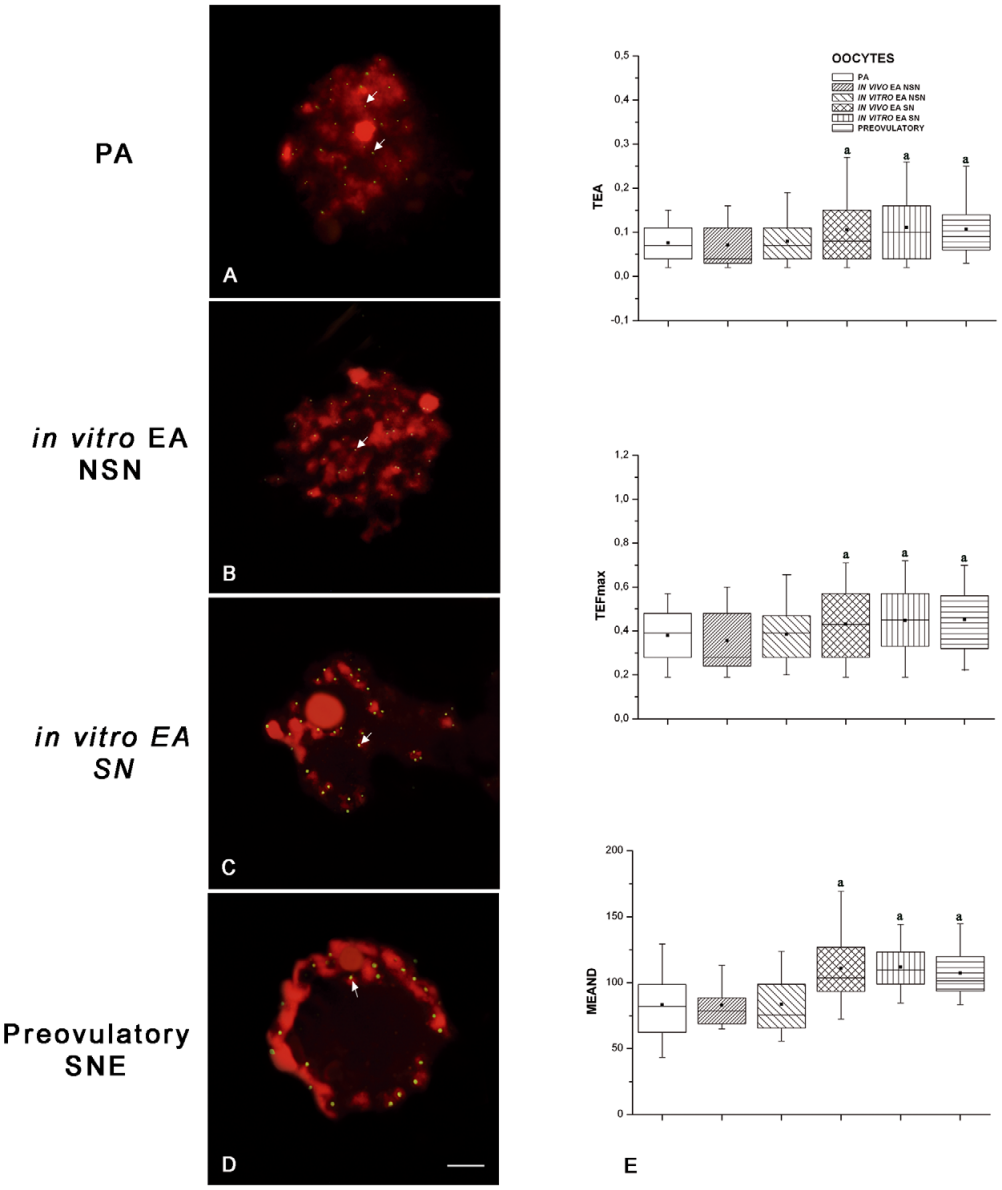
Germinal vesicle (GV) telomeres analyzed using the Q-FISH technique. Examples of paraffin-embedded follicle sections with telomeres visualized as bright green spots (FITC), while DNA was counterstained in red with PI. On the left panel digital images of FISH for telomeres are shown (A–D), on the right panel (E) quantitative analyses of telomeres were performed in the GVs of all *in vitro* and *in vivo* classes of germ cells analyzed. (**A**) Oocyte derived from a preantral (PA) follicle showing a not surrounded nucleolus (NSN) chromatin configuration and the relative telomeres. (**B,C**) GV comparison between *in vitro* grown oocytes isolated from early antral (EA) follicles displaying a NSN and surrounded nucleolus (SN) chromatin configuration: the images reveal a higher fluorescence intensity for telomeres in SN oocytes. (**D**) Oocyte derived from a preovulatory follicle that displayed telomeres close to the condensed chromatin distributed around the nucleolus and the nuclear membrane (SNE configuration). In all the digital images, telomeres (arrows) are distributed in correspondence to the chromatin. Scale bars = 5 µm (**E**) Telomere area (TEA), feret maximum (TEFmax), and mean densitometric value (MEAND) examined in the GVs: the horizontal lines in the box plots express the 5^th^, 25^th^, 50^th^, 75^th^, and 95^th^ percentile of the distribution; the box stretches from the 25^th^ to the 75^th^ percentile, and therefore contains the middle half of the scores in the distribution; the median is shown as a line across the box, meanwhile the mean value as a black square within the box. The data of TEA, TEFmax, and MEAND that resulted highly significantly different (*P*<0.01) after statistical analysis were indicated by the superscript ^(a)^.

The Fig. 6 shows as telomere length was affected by oocyte diameter and chromatin configuration but not influenced by culture. Telomeres in the oocytes isolated from PA follicles had an area (TEA) of 0.07±0.04, a feret maximum (TEFmax) of 0.37±0.12, and a mean densitometric value (MEAND) of 83.2±27.16 (Fig. 6). Telomere size increased in all the oocytes isolated from EA follicles that had developed the SN chromatin configuration (*P*<0.01; Fig. 6). By contrast, NSN oocytes isolated from *in vitro* grown EA follicles maintained telomere parameters similar to those recorded in PA oocytes (*P*>0.05; Fig. 6). The process of telomere elongation stopped in germ cells enclosed in antral follicles (Fig. 6).

#### E. Oocyte tert intracellular distribution

**Figure 7:**
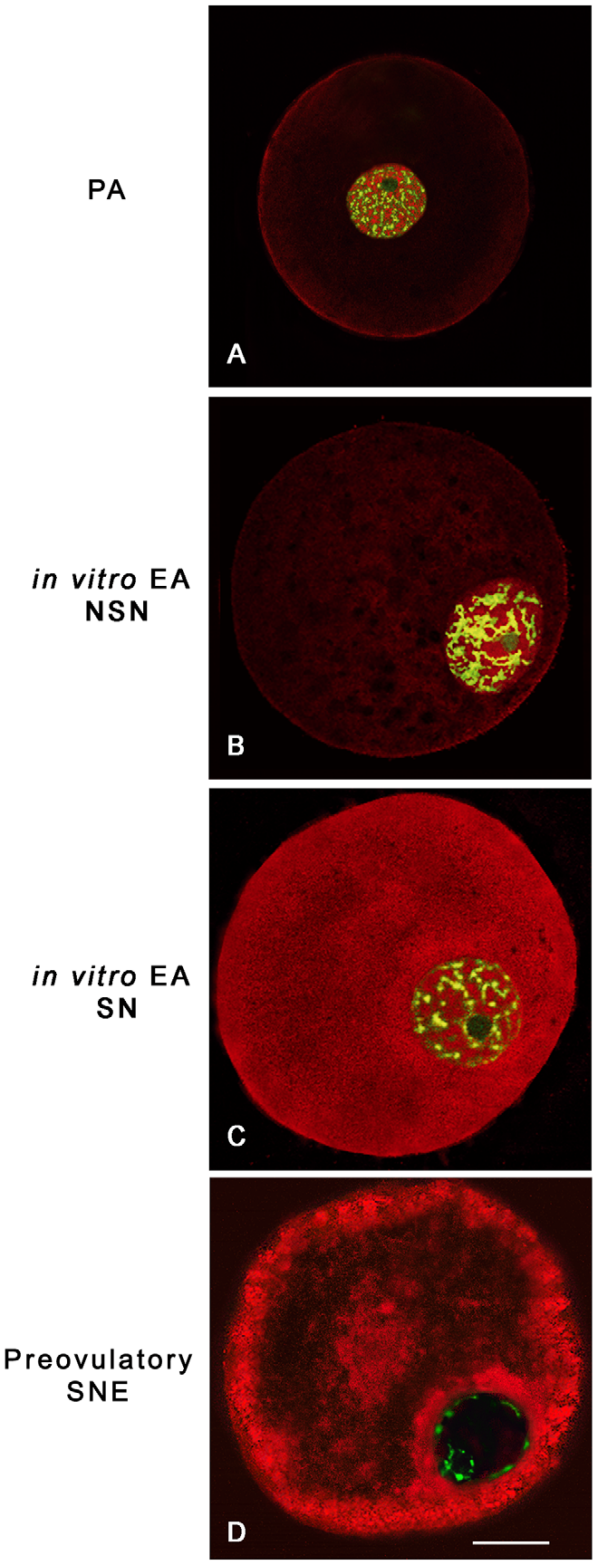
Oocyte distribution of the telomerase catalytic subunit (TERT). Examples of oocyte TERT distribution, visualized with Cy3-conjugated secondary antibody (red), while DNA was counterstained in green with SYBR Green 14/I. TERT sub-cellular localization was influenced by chromatin configuration in all *in vitro* and *in vivo* classes of germ cells analyzed. (**A,B**) Oocytes derived from a preantral (PA) follicle (A) and an *in vitro* grown early antral (EA) follicle (B) with a not surrounded nucleolus (NSN) chromatin configuration which localized TERT in the nucleus. (**C**) *in vitro* grown oocyte derived from an EA follicle with a surrounded nucleolus (SN) chromatin configuration which reveals TERT between the nucleus and ooplasm. (**D**) Oocyte of a preovulatory follicle with condensed chromatin localized surrounding the nucleolus and the nuclear envelope (SNE configuration) and with a clear subcortical and perinuclear cytoplasmatic TERT distribution. Scale bar = 25 µm.

TERT intracellular distribution was influenced by chromatin configuration as indicated in Fig. 7. In fact, TERT was localized in the nucleus of NSN oocytes (Fig. 7) and between nucleus and cytoplasm in SN oocytes isolated from EA follicles (Fig. 7). The enzyme became exclusively cytoplasmatic with a subcortical and perinuclear distribution in oocytes isolated from preovulatory follicles (Fig. 7).

#### F. Methylation pattern at single gene level

The DNA methylation status at the differentially methylated regions (DMRs) of two maternally (*IGF2R* and *BEGAIN*) and one paternally (*H19*) imprinted genes was determined in:

- GV oocytes isolated from PA, *in vitro* and *in vivo* grown EA, and preovulatory follicles;
- metaphase II (MII) oocytes obtained after *in vivo* maturation, and *in vitro* maturation (IVM) of *in vitro* and *in vivo* grown EA follicles and medium antral follicles.

In all oocytes analyzed, the 5′-end DMR of *H19* maintained a no methylated status (data not shown).

**Figure 8:**
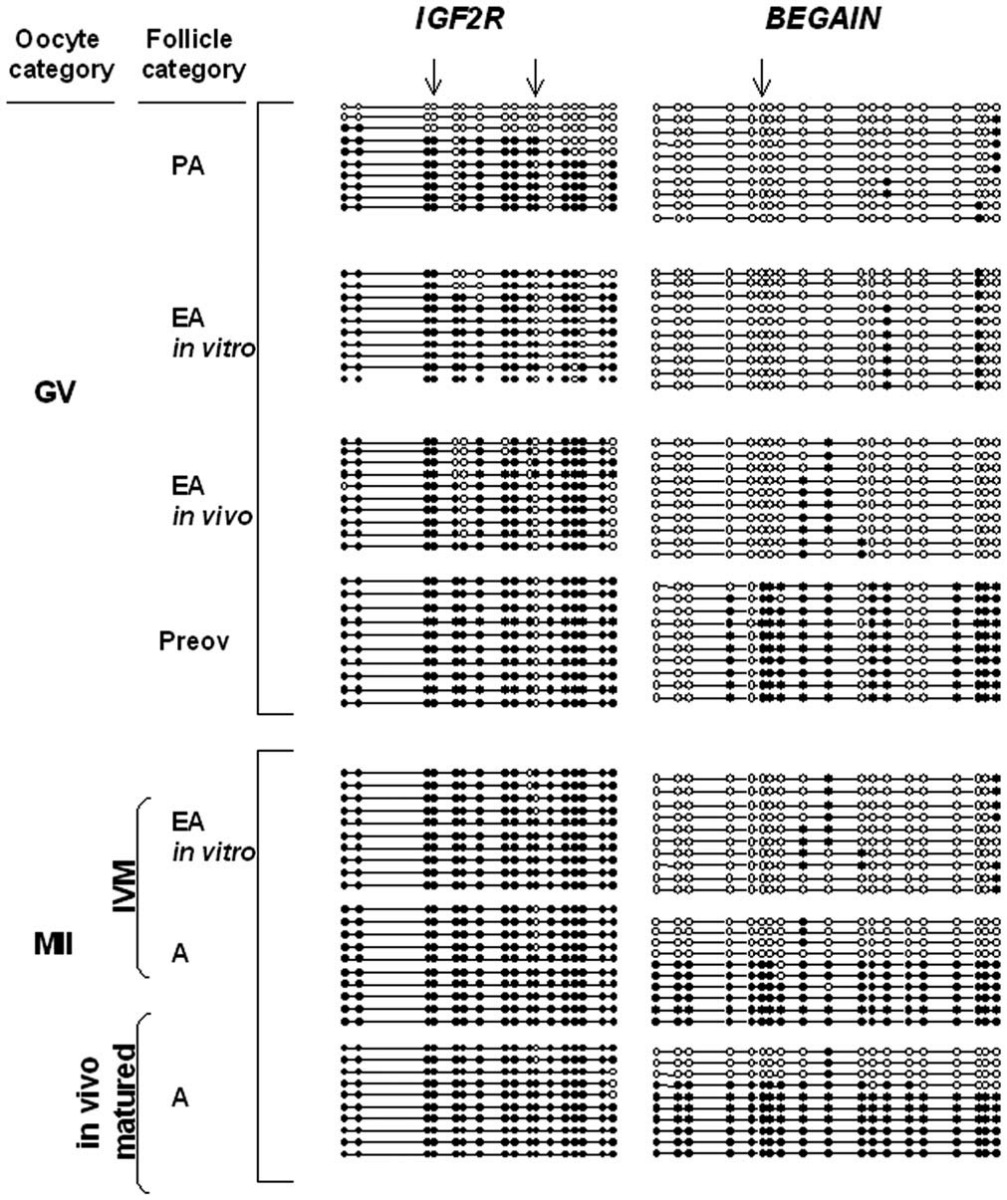
Methylation status of the *IGF2R* and *BEGAIN* genes in germ cells analyzed by bisulfite sequencing. An example of bisulphite sequencing analysis of examined CpGs in three imprinted genes. Each line represents an individual sequence molecule, with each circle corresponding to a separate CpG site (CpG). Black and white circles indicate methylated and unmethylated CpGs respectively. Arrows indicate the CpGs assayed by *BstUI* and *HhaI* enzymes, used in the combined bisulfite restriction analysis (COBRA). GV, germinal vesicle; MII, metaphase II; IVM, *in vitro* maturation; PA, preantral follicles; EA, early antral follicles; Preov, preovulatory follicles; A, antral follicles. Percentages of methylation were calculated by counting the number of methylated CpGs out of the total number of CpGs within the fragment amplified.

Consistently with our previous results [33], GV oocytes isolated from PA follicles showed ∼5% and 55% of methylated CpG sites (CpGs) in the exon 9 DMR of *BEGAIN* and in the DMR2 of *IGF2R*, respectively (Fig. 8). The *in vitro* and *in vivo* EA enclosed grown oocytes increased the *BEGAIN* and *IGF2R* methylation status that became ∼10% and ∼85%, respectively (Fig. 8). The degree of methylation recorded in GV oocytes collected from preovulatory follicles was stable for *IGF2R* (∼90%), while resulted significantly higher for *BEGAIN* (∼60%).

Resumption of meiosis did not affect the methylation status of *BEGAIN* and *IGF2R:* in MII oocytes obtained from *in vivo* (data not shown) and in *in vitro* grown oocytes isolated from EA follicles the percentage of the methylated sites remained unchanged (∼10% and ∼90%, respectively) and resulted similar to that of MII oocytes collected from the oviduct of hormonal primed animals (∼60% and ∼90%, respectively; Fig. 8).

**Figure 9:**
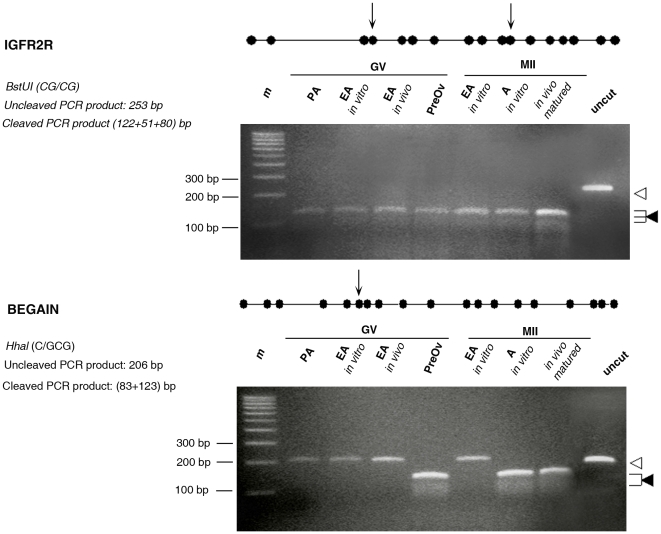
Overall methylation status of the *IGF2R* and *BEGAIN* genes in germ cells analyzed by COBRA. The same DNA amplified by PCR and used for bisulfite sequencing was digested by enzymes that cut only if the site was methylated. The size of the expected fragments for the unmethylated (<) and methylated (◂) alleles are indicated. The undigested and digested products are indicative of unmethylated and methylated templates respectively. Combined bisulfite restriction analysis (COBRA) showed on overall methylation pattern comparable to the one obtained by bisulfite sequencing. In those samples in which there are both methylated and unmethylated clones (e.g. GV and PA for *IGF2R* and MII oocytes for *BEGAIN*) the specific restriction enzyme cutting sites are preferentially methylated, resulting only in cleaved products after enzymatic digestion. m = 100 bp Marker; uncut = undigest PCR product. Cleavage CpG sites (CpGs) are indicated: *BstUI* (CG/CG) cuts between CpGs 3 and 4, 10 and 11 in *IGF2R*. *HhaI* (C/GCG) cuts at CpG 6 in *BEGAIN*. Preov, preovulatory follicles isolated from hormonal treated sheep.

To confirm that the sequencing results accurately reflected the overall methylation pattern, combined bisulfite restriction analysis (COBRA) was carried out on all samples. The *H19* DMR was shown to yield the predicted uncleaved band after *BstUI* digestion, confirming that the analyzed region was completely unmethylated in all classes of female gametes and that there was no contamination with somatic cells (data not shown). The COBRA assay carried out for *IGF2R* and *BEGAIN* DMRs also showed comparable results with those obtained from bisulfite sequencing (Fig. 9). Digestion of the *IGF2R* and *BEGAIN* PCR products with *BstUI* and *HpaI* enzymes resulted in the production of cleaved and uncleaved fragments, since only a limited number of CpGs can be assayed using the restriction-based method (Fig. 9). Overall, restriction analysis showed a good percentage of data concordance with the sequencing results.

### EVALUATION OF OOCYTE COMPETENCE

#### A. Meiotic competence

As showed in Table 2, co-*in vitro* maturation (co-IVM) did not promote meiosis in oocytes isolated from PA follicles. On the contrary, ∼45% of *in vitro* and *in vivo* grown oocytes isolated from EA follicles reached the MII stage at the end of the co-IVM. In both the categories of oocytes, however, the percentage of MII oocytes resulted significantly lower than that of the oocytes isolated from medium antral follicles (∼95%; *P*<0.01).

**Table 2:** Ooocyte nuclear stage recorded in different categories of oocytes after IVM.

Follicle category	Analyzed oocytes (n°)	Degenerated (%)	Nuclear stage of *in vitro* matured oocytes (%)		
			GV	GVBD/MI	MII
***In vivo*** ** grown**					
PA	55	14±3^a^	96±4^a^	4±1^a^	----
EA	88	11±2^a^	35±6^b^	17±3^b^	48±9^a^
A	205	5±1^b^	2±1^c^	3±1^a^	95±12^b^
***In vitro*** ** grown**					
EA	110	19±4^c^	44±11^b^	15±3^b^	43±11^a^

Values are the means ± s.d. Different letters within each column indicate statistically significant differences (*P*<0.01, ANOVA test). n°, total number of oocytes analyzed; the number of degenerated oocytes was subtracted from the total number. GV, germinal vesicle; GVBD/MI, germinal vesicle breakdown/metaphase I; MII, metaphase II; PA, preantral follicles; EA, early antral follicles; A, antral follicles.

#### B. Parthenogenetic development

In order to determine the degree of cytoplasmic maturation, parthenotes were produced by activating the previous classes of MII oocytes. As showed in Table 3, ∼60% of oocytes isolated from medium antral follicles resulted activated while ∼40% of them underwent more than 4 divisions in 96 hours. Parthenogenic activation of oocytes isolated by EA follicles produced a lower percentage of embryos (40–50%) and only a 10% of them with more of 16 blastomeres, regardless of the category considered.

**Table 3:** Parthenogenetic activation of MII oocytes obtained from different categories of follicles.

Follicle category	Analyzed oocytes (n°)	Degenerated (%)	Developmental stage reached by healthy activated oocytes after 96 h (%)		
			Uncleaved	<16 cells	>16 cells
***In vivo*** ** grown**					
A	300	4±1^a^	38±3^a^	19±2^a^	43±5^a^
EA	150	9±1^b^	52±9^b^	38±6^b^	10±2^b^
***In vitro*** ** grown**					
EA	150	12±3^b^	61±14^b^	34±9^b^	5±3^b^

Values are the means ± s.d. Different letters within each column indicate statistically significant differences (*P*<0.01, ANOVA test). n°, total number of oocytes analyzed. The degenerated oocytes were subtracted from the total number of oocytes. EA, early antral follicles; A, antral follicles.

#### C. Early embryo development after ivf

As showed in Table 4, the fertilization rate of oocytes isolated from medium antral follicles was significantly higher (∼64%) than that recorded in MII oocytes obtained from *in vitro* and *in vivo* grown EA follicles (∼32 and 40%, respectively; *P*<0.01). Moreover, the IVM/IVF oocytes derived from medium antral follicles reached a more advanced stage of embryo development (38% with more than 16 blastomeres) than that of oocytes isolated from *in vitro* and *in vivo* grown EA follicles (∼10 and 13%, respectively) after 6 days of culture.

**Table 4:** Early embryo development of IVM/IVF oocytes derived from *in vitro* and *in vivo* grown follicles.

Follicle category	Analyzed oocytes (n°)	Fertilization rate (%)	Embryonic stage 6 days after IVF (%)	
			<16 cells	>16 cells (n° early blastocyst)
***In vivo*** ** grown**				
A	400	64±7^a^	62±12^a^	38±7^a^ (14)
EA	325	40±12^b^	84±18^b^	13±3^b^ (2)
***In vitro*** ** grown**				
EA	336	32±9^b^	91±12^b^	10±2^b^(1)

Values are the means ± s.d. Different letters within each column indicate statistically significant differences (*P*<0.01, ANOVA test). n°, total number of oocytes analyzed. EA, early antral follicles; A, antral follicles.

## Discussion

Following early studies in the mouse, the present research demonstrates that the complex processes involved in early tertiary phase of follicle/oocyte growth can be successfully reproduced *in vitro* also in a medium-sized animal like the sheep.

The factors underlying secondary to tertiary follicle transition are still poorly understood, but in our *in vitro* system, gonadotropin stimulation appeared to be sufficient to drive a coordinated follicle and oocyte development [34]–[36]. In fact, a high percentage of PA follicles (70%) reached the EA stage, showing a tissue organization and an oocyte development similar to that observed in EA follicles obtained from sheep ovaries immediately after collection. The presence and mainteinance of the theca layer in our culture system may represent a key condition for the achievement of these results and may explain the shortened time of culture required to produce EA follicles in comparison to Newton et al. [37]. The presence of the theca layer allows, in fact, to use media supplemented with serum that otherwise must be omitted to avoid that follicular cells could adhere to the culture surface. In addition, theca cells may better contribute to follicle/oocyte growth either producing stimulating paracrine factors or by conserving a physiological three dimensional follicle organization [38], [39]. However, to finally respond to this hypothesis a comparative study of intact and theca-free PA follicles must be undertaken but this is not the major point of the current work.

A finding of particular interest was that oocytes underwent physiological step-wise chromatin changes under this favourable cultural system over a period of 14 days.

In fact, the degree of global DNA methylation, telomere elongation and TERT/Dnmt3a distribution were equivalent in oocytes grown under *in vitro* and *in vivo* conditions. In addition, after *in vitro* oogenesis the germ cells increased significantly their ability to undergo meiosis and early embryo development, thus displaying functional performances equivalent to those expressed by *in vivo* grown oocytes isolated from EA follicles.

However, the *in vitro* folliculogenesis anticipated the nuclear export of the Dnmt1 and induced in a low percentage of oocytes the SN chromatin configuration without negatively affecting their early embryonic developmental competence.

Many reports proposed, on the contrary, a positive relationship between the SN chromatin configuration and oocyte meiotic and developmental competence in several species [9], [10], [40]. In sheep, the *in vitro* grown oocytes displaying a lower percentage of SN chromatin configuration did not show a different incidence of MII oocytes and early embryos that in *in vivo* grown oocytes derived from EA follicles.

In addition, the anticipated Dnmt1 nuclear export did not influence DNA methylation either at a global or single gene level. During the transition from secondary to tertiary follicles, global DNA methylation proceeded either under *in vitro* or *in vivo* conditions reaching similar levels. Fortunately, the process of global DNA methylation is not temporally associated to chromatin condensation but resulted, on the contrary, strictly correlated to the process of oocyte growth. The *in vitro* grown germ cells, in fact, reached a diameter of 110±5 µm thus acquiring a global DNA methylation (TFI value) that was similar to that recorded in the oocytes isolated from all the categories of tertiary follicles (EA and preovulatory follicles). The DNA methylation status at single gene loci seems to confirm a correct *in vitro* evolution of the DNA methylation process, even if the exit of Dnmt1 from the nucleus was anticipated. In fact, the methylation status of an early (*IGF2R*) and a late sheep imprinted gene (*BEGAIN*) was reproduced in culture with a schedule similar to that described under physiological conditions [33]. In fact, it has been recently demonstrated that *BEGAIN* acquired an almost complete methylation imprint at its exon 5 DMR in late stages, while *IGF2R* gene showed an overall degree of methylation, in all CpG islands within its DMR2, already in the early stages of oocyte growth. Under *in vitro* culture the process of *IGF2R* gene methylation was completed in oocytes collected by EA follicles, while *BEGAIN* methylation displayed a modest rise. This results is, however, in agreement with its late imprinting definition [33]. In fact, *BEGAIN* methylation physiologically reached high levels exclusively in GV oocytes collected from late A follicles (medium sized or preovulatory follicles) and it was not affected by the resumption of meiosis [33]. In the present paper even if the levels of methylation reached in oocytes grown *in vitro* are lower than those of oocytes that are usually used in ART procedures they resulted equivalent to those recorded in GV/MII oocyte isolated from *in vivo* grown EA follicles.

The combination of these results with the kinetic of Dnmt1 nuclear export indicated that this enzyme did not have any role in maternal genome imprinting establishment after the transition from secondary to tertiary follicle. During this late stage, Dnmt1 did not have any access to the genome since the protein was confined into the cytoplasm of all the oocytes. The unique process that was temporally correlated with the nuclear presence of Dnmt1 under *in vitro* and *in vivo* conditions is the process of global DNA methylation, as previously supposed [41], [13]. By contrast, the nucleus distribution of Dnmt3a in the oocytes monitored during the *in vitro* and *in vivo* oogenesis may suggest a role of this remodelling DNA enzyme in maternal genome imprinting. As indicated by other groups [42], [43] and confirmed by the methylation profile of *BEGAIN* [33], the epigenetic mechanisms of maternal imprinting remain operative until the later stage of oocyte differentiation, thus indicating the selective involvement of Dnmt3a that maintained an interaction with the DNA until the resumption of oocyte maturation. In addition also functional experiments confirm these hypothesis: offspring from Dnmt3a conditional mutants mice females die in utero displaying lack of methylation and allele-specific expression at several maternally imprinted loci [44].

The analysis of the methylation status at single gene level, moreover, showed as under the present long term *in vitro* culture the epigenetic program in growing oocyte was not negatively influenced.

Differently from two previous reports [30], [45], in fact, the processes of *in vitro* growth and maturation in sheep oocytes occurred with a correct methylation profile of I*GF2R* and *BEGAIN* and with a stable unmethylated status of the paternally imprinted *H19* gene. These data confirm that the cultural conditions adopted here were able to actively support the process of epigenetic remodelling without inducing any abnormalities previously ascribed to the medium composition or to the protracted period of incubation [30], [45].

As described in mouse and human, another nuclear parameter strictly related to the developmental competence of oocytes is represented by telomere length [46]–[48]. This process of telomere elongation resulted particularly intense during the transition from PA to EA follicles, thus becoming a crucial step-wise nuclear change to monitor in order to evaluate the process of *in vitro* oocyte growth [13]. The present research, with the aid of the quantitative-fluorescence in situ hybridisation (Q-FISH) technique, which allows to assess telomere size on single germ cells [49]–[52], highlights that telomeres are actively rearranged during sheep oocyte growth obtained under *in vitro* and *in vivo* condition. In particular, telomere elongation stopped in SN germ cells isolated from EA follicles. Fortunately, TERT nuclear localization was prolonged in NSN oocytes isolated from *in vitro* grown EA follicles, thus probably guaranteeing the persistence of the process of telomere elongation in these immature chromatin category of oocytes produced *in vitro*. The evidence of different telomere elongation in oocytes belonging to the categories of *in vitro* grown oocytes (NSN and SN oocytes) could explain their slightly reduced developmental competence compared to that recorded in EA *in vivo* grown oocytes.

The comparative analysis of telomere length in parthenogenetic embryos derived from *in vitro* and *in vivo* grown oocytes isolated from EA follicles is now under progress in order to confirm the positive correlation between telomere size and early embryo developmental outcome in sheep.

This hypothesis has been recently proposed by [47] that demonstrated as a reduced telomere length of embryos derived from gametes collected from women undergoing IVF treatment was associated with a higher incidence of cytoplasmic fragmentation and apoptosis. If this new experimental approach will confirm a strict positive correlation between telomere length and embryos development, the evaluation of the telomere length could be proposed to predict the outcome of reproductive programs.

On the basis of the above results, it can be concluded that i*n vitro* folliculogenesis here adopted for sheep PA follicles is able to guarantee nuclear/chromatin events that drive the oocyte to reach a functional asset compatible with the oocyte/follicular stage reached. Unfortunately, the process of oocyte differentiation is extremely slow in medium sized mammals and the degree of meiotic maturation in EA oocytes is quite incomplete if compared to the mouse. In fact, as previously described [53] the gamete isolated from sheep small antral follicles showed a very low meiotic competence due to an immaturity of both the somatic and the germ compartment. In fact, a very low percentage of sheep oocytes collected from EA and small antral follicles [54], [55] underwent meiosis, in part, for the inability of cumulus cells to trigger maturation after gonadotropin stimulation [32]. However, as demonstrated recently the immaturity of cumulus cells surrounding the oocyte isolated from sheep small antral follicles, can be overcome by introducing in the culture system soluble stimulating factors derived by fully competent cumulus-oocyte complexes [52]. The prerequisite condition for these soluble stimulating factors to induce meiosis is represented by the presence of a functional communication between cumulus cells and the oocyte. For this reason, the similar maturation capacities of oocytes obtained in *in vitro* and *in vivo* grown oocytes could be interpreted as an indirect confirmation that folliculogenesis performed in culture was able to preserve a constant metabolic coupling between germinal and somatic compartments.

However, in medium sized mammals the oocyte needs to progress through the late tertiary follicle stage to acquire a complete developmental competence, thus indicating that further efforts need to be adopted in order to define a conclusive *in vitro* folliculogenesis protocol. An improvement of this cultural system will be tax from the incomplete genomic imprinting (e.g. *BEGAIN*) which represents a vital prerequisite for the success of both fetal and adult life [56].

In this context, all the studies, like this, performed in order to evaluate the effect of the *in vitro* folliculogenesis technique on the process of oogenesis may be particular useful by representing an instrument to increase the knowledge on the complex mechanisms leading oocyte differentiation, in particular, when the investigation involved a translational mammal model.

## Materials and Methods

### ETHICS STATEMENT

The present animal work has been conducted in strict accordance with relevant national and international guidelines. The authors have the permission of the Italian Ministry of Health to conduct animal experimentation (ovariectomy and tubal flushing in ewes performed under general anaesthesia, with no suffering) according to the consent by silent contemplated by Italian law D.Lgs. 116/92, thus no permit/approval number was needed. The animals were not sacrificed and regained their production cycle.

### CHEMICAL AGENTS

All chemical reagents and media used for the *in vitro* culture were purchased from Sigma Chemical Co (St Louis, MO, USA), unless otherwise specified.

### FOLLICLE AND OOCYTE COLLECTION

Prepubertal sheep ovaries were obtained from the local abattoir (Mattatoio Comunale Cooperativa Macellatori Teatini) and transported to the laboratory in a thermostatic container within 60 minutes from collection. Ovaries were rinsed several times in Phosphate Buffer Saline (PBS) supplemented with antibiotics (75 mg/l penicillin-G, 50 mg/l streptomycin sulphate), transferred into HEPES-buffered TCM199 medium, and cut into cortical fragments (0.5×0.5×0.5 cm). PA and EA follicles were mechanically isolated from cortical fragments and selected on the basis of their morphology and size [31] in order to maintain intact the theca layer (Fig. 1). PA follicles used for *in vitro* culture experiments displayed a mean diameter of 170±30 µm [13]. The PA follicles were accurately measured under an inverted-phase microscope equipped of an ocular micrometer (40× magnification) in order to exclude from the culture EA follicles and to use PA follicles with higher *in vitro* performance as previously demonstrated either from our [31], [32] and Newton group (PA cumulus-oocyte complexes 190–240 µm) [37]. Morphology was assessed before and after the culture by fixing the follicles in 4% paraformaldehyde/PBS for 12 h at 4°C. After dehydration, each tissue sample was embedded in paraffin wax, serially sectioned at 5 µm thickness and stained with haematoxylin and eosin, as previously described [32] (Fig. 1).

For the comparative analysis GV oocytes were also collected from:

1. EA follicles displaying a mean diameter of 360±60 µm,
2. preovulatory follicles (>6 mm) surgically isolated from hormonal treated sheep [57], [58],

and MII oocytes collected from:

1. surgically flushed oviducts of hormonal treated sheep [59]
2. IVM cumulus-oocyte complexes collected from medium sized A follicles.

Surgery was always performed maintaining the animals under anesthesia with acepromazine maleate (0.05 mg/kg bodyweight) and pentothal sodium (10 mg/kg bodyweight).

### *IN VITRO* FOLLICLE GROWTH

PA follicles were individually cultured in 96-V-well microtiter plates in 25 µl of α-MEM supplemented with 2% fetal calf serum (FCS), 1% insulin, transferrin, and selenium (ITS), antibiotics (75 mg/l penicillin-G, 50 mg/l streptomycin sulphate) and 1 µg/ml ovine FSH (oFSH) under pre-equilibrated mineral oil. The culture were maintained for 14 days at 38.5°C in gas mixture (5% O_2_, 5% CO_2,_ 90% N_2_) within a humidified incubator. Culture medium was changed every two days. Each follicle were measured under a stereomicroscope at the beginning and at the end of the culture. Only those showing the presence of an antral cavity were chosen for the following experiments. The enclosed oocyte-cumulus cell complexes (OCCs) were recovered from mechanically opened follicles. Complexes showing continuous and compact layers of cumulus cells were then utilized.

### ANALYSIS OF CHROMATIN CONFIGURATION IN GV OOCYTES

GV oocytes recovered from PA, *in vitro* and *in vivo* grown EA and preovulatory follicles were measured after cumulus cells and zonae pellucida removal [59]. Zona-free oocytes were fixed in 4% paraformaldehyde/PBS for 1 hour at 4°C. To analyze chromatin organization, at least 40 oocytes for each follicle category and 5 oocytes from preovulatory follicles were labelled for 30 minutes with 1 µM SYBR Green 14/I (Molecular Probes, Eugene, OR, USA), and then analyzed with a laser scanning confocal (LSC) microscope as described below.

### ASSESSMENT OF GLOBAL DNA METHYLATION

According to [13], fixed oocytes were incubated with mouse anti-5-methylcytosine antibody (1∶500 dilution; Eurogentec, San Diego, SA, USA), and, then, with a secondary goat anti-mouse antibody Cy3-coniugated (1∶400 dilution; Molecular Probes). The oocytes were counterstained with 1 µM SYBR Green 14/I. Negative control was always performed by using non-immune serum in place of the primary antisera. Immunofluorescence analyses were carried out on at least 40 oocytes for each follicle category considered, and on at least 20 oocytes isolated from preovulatory follicles by LSC microscope.

### LOCALIZATION OF DNMTs AND TERT

Oocyte Dnmt1 and Dnmt3a localization was assessed using a polyclonal goat antibody anti-Dnmt1 (1∶200 dilution; Santa Cruz Biotechnology, Santa Cruz, CA, USA) [13], and a polyclonal rabbit antibody anti-Dnmt3a (1∶100; Abcam, Cambridge, UK). A secondary donkey anti-goat Alexa Fluor488 conjugated antibody (1∶400; Molecular Probes) was used for Dnmt1, and a goat anti-rabbit conjugated Cy3 antibody (1∶400; Molecular Probes) for Dnmt3a detection. Nuclear counterstaining was carried out with 1 mg/ml Propidium iodide (PI) or 1 µM SYBR Green 14/I.

The distribution of telomerase catalytic subunit was assessed according to [13] by using a polyclonal rabbit antibody anti-telomerase reverse transcriptase (anti-hTERT; 1∶250; Calbiochem, Merck KGaA, Darmstadt, Germany). A goat anti-rabbit conjugated Cy3 (1∶400; Molecular Probes) was used as secondary antibody, while chromatin was counterstained with 1 µM SYBR Green 14/I.

In all experiments non-immune serum was used in place of the primary antisera as a negative control. Each immunofluorescence analysis was carried out on at least 40 oocytes for each follicle category, and on at least 10 oocytes isolated from preovulatory follicles by LSC microscope.

### LASER CONFOCAL MICROSCOPY ANALYSIS

Observations of different fluorescence probes were performed with a Bio-Rad laser scanning confocal microscope (Radiance 2000 IK-2, Hemel Hempstead, UK), equipped with a krypton/argon ion laser. The samples were analyzed on an inverted microscope (Zeiss Axiovert, Oberkochen, Germany) equipped with a plan-apochromat oil immersion objective 63× magnification/1.4 numerical aperture (NA). Immunostained oocytes were observed using the visible lines of excitation of 488 nm and 568 nm, and a dichroic filter (560LP, Bio-Rad). Digital optical sections were obtained by scanning the sample on z-axis at 0.2 µm of thickness throughout the plane of focus containing the GV equatorial plane (±20 µm). For each experiment all the categories of oocytes were compared maintaining similar gain and laser parameters.

The degree of global DNA methylation was evaluated according to [59]. In brief, the z-series obtained were merged to produce a two-dimensional image showing the staining pattern and the TFI emitted by each GV. The TFI emitted from each GV was measured using the LaserPix software (Bio-Rad).

### TELOMERE SIZE DETECTION BY FLUORESCENCE IN SITU HYBRIDISATION (Q-FISH)

Five-micron thick sections, obtained from paraffin embedded follicles belonging to all groups considered, were processed for FISH as described in [13], [52]. A denatured all-human telomeric DNA probe with conserved non-coding sequences of DNA repeats (TTAGGG)_n_. [60] was used. The hybridized signals were detected by using a commercial kit (FITC-avidin detection kit; Oncor Inc, Gaithersburg, MD, USA) according to the manufacturer's instructions. PI/Antifade 0.6 µg/ml (Qbiogene – Resnova, Roma, Italy) was used for chromatin counterstaining. All slides were analyzed using an Axioskop 2 Plus incident-light fluorescence microscope (Zeiss).

Quantification of telomere length was performed according to [13], [52]. For each oocyte, at least 2 cross sections which included the nuclei of the oocytes were considered. Q-FISH analysis was performed on at least 20 oocytes for each category of follicles. In brief, for quantitative purposes digital images were consecutively captured from sections immediately after hybridization. At the beginning of an imaging session, optimum exposure times were determined and held constant thereafter. The oocyte GV were acquired by setting the luminous-field diaphragm on the whole area of the nucleus. This procedure was performed in order to exclude from the acquisition the fluorescent signals of granulosa cells. In all cases, it was confirmed that the telomeric signals were within the linear response range of the CCD camera using standard fluorescent microbeads (InSpeck microspheres, Molecular Probes). Image acquisition was performed with a dedicated software (Axiovision, Zeiss). After the acquisition of the digitized images, they were processed, using the dedicated computer program (KS300 computed image analysis system, Zeiss).

Quantification of the digitized fluorescent telomere signals was accomplished after densitometric calibration of the background, the algorithm performs: (i) segmentation of telomeres; (ii) measurements of their areas, lengths, and MEAND. Parameters to be measured were then selected; these included MEAND, area (TEA), and feret maximum (TEFmax), which corresponds to the value of the major diagonal connecting the two farthest points at the periphery of the object. Results were then recorded for statistical evaluation.

### BISULFITE SEQUENCING

Genomic DNA was isolated from three pools of about 50 oocytes for each groups of follicles as previously described [33]. In detail, DNA isolation was performed using a Chelex resin-mediated protocol [33] and bisulfite mutagenesis according to [61].

All mutagenized DNAs were subjected to three independent nested or semi-nested PCR amplification, as previously described [33], to eliminate the sampling bias of PCR. Each bisulfite-treated DNA was amplified using primers for specific DMRs of *IGF2R* (GenBank accession number: AY182033), *BEGAIN* (GenBank accession number: AY509925) and *H19* (GenBank accession number: AJ566210) genes, as previously described by [33]. The PCR samples were subcloned using the pGEM-T Vector System II (Promega, Madison, WI, USA) and bacterial clones containing the appropriate inserts were sequenced using an ABI 310 sequencer (Applied Biosystem, Forster City, CA, USA). Twenty clones for each sample were sequenced on average and only sequences derived from clones with more than 95% cytosine conversion were analyzed. Percentages of methylation were calculated by counting the number of methylated CpGs out of the total number of CpGs within the fragment amplified. To exclude eventual clonality of sequences, clones with similar CpG methylation patterns were compared by evaluating the C→T conversion at non-CpG sites.

### COMBINED BISULFITE RESTRICTION ANALYSIS (COBRA)

Approximately 200 ng of the same bisulfite-treated PCR products used for cloning and sequencing were digested with *BstUI* (*IGF2R*, *H19*) or *HhaI* (*BEGAIN*) (New England Biolabs, Ipswich, MA, USA). Since both enzymes cleave only the methylated templates at CG/CG and C/GCG restriction sites, the digestion of PCR products is indicative of methylated CpGs. Digestion results were analyzed by 3% agarose gel electrophoresis containing ethidium bromide and visualization on a UV transilluminator.

### *IN VITRO* MATURATION

The OCCs isolated were matured *in vitro* (IVM) as previously described [53]. In detail, 30 OCCs collected by medium sized antral follicles (3–4 mm in diameter), able to progress in meiosis in high percentage [55], were co-cultured (co-IVM) with 15 OCCs isolated from PA or from *in vitro* or in *vivo* grown EA. The co-IVM were performed in 60 µl TCM199 medium containing 2 mM glutamine, 100 µM cysteamine, (0.3 mM sodium pyruvate), 5% FCS and gonadotropins (0.1 UI/ml ovine FSH and/or 0.1 UI/ml porcine LH, USDA-pLH-B-1; National Institute of Diabetes and Digestive and Kidney Diseases, National Institutes of Health, Bethesda, MD, USA) in 96-well flat-bottom plates (NUNC, Rochester, NY, USA). IVM was carried out under mineral oil for 24 hours at 38.5°C in 5% CO_2_ in air with 100% of humidity. At the end of IVM, OCCs were denuded from surrounding cumulus cells and analyzed under a stereomicroscope for the presence of the extruded polar body (MII oocytes). The oocytes that reached the MII stage were tested for their developmental competence or used for bisulfite mutagenesis analysis.

### *IN VITRO* EMBRYO PRODUCTION

The developmental competence of different categories of MII oocytes were tested either by parthenogenesis or after IVF. Mammalian parthenotes were studied in order to evaluate the exclusive roles of maternal cytoplasm/genomes in early mammalian development.

Parthenogenetic activation was carried out on matured denuded oocytes according to [62]. After 96 hours from activation, both uncleaved and cleaved oocytes were fixed, and then stained with 1% Lacmoid [62] to evaluated their developmental stage by counting the numbers of blastomeres. IVF was performed with frozen-thawed semen, obtained from rams of proven fertility. In brief, fertilization was carried out in 50 µl drops, at a concentration of 1×10^6^ sperm/ml, at 38.5°C in a humidified atmosphere of 5% CO_2_ in air for 20–22 hours. After 6 days of culture, uncleaved and cleaved eggs were fixed, stained with Lacmoid [62] and analyzed.

### STATISTICAL ANALYSIS

The normalcy of data was tested with Shapiro-Whilks test before proceeding with ANOVA test, followed, when necessary by the post hoc Tukey test (Microcal Origin 6.0). The data were expressed as mean ± standard deviation (s.d.). Statistical analyses were carried out on transformed data by arctan(x). This transformation has been used to more closely meet the conformity to Gaussian distribution of data. The data were considered significant and highly significant for *P*<0.05 and *P*<0.01, respectively.
